# Learning to explain is a good biomedical few-shot learner

**DOI:** 10.1093/bioinformatics/btae589

**Published:** 2024-10-03

**Authors:** Peng Chen, Jian Wang, Ling Luo, Hongfei Lin, Zhihao Yang

**Affiliations:** School of Computer Science and Technology, Dalian University of Technology, Dalian 116024, China; School of Computer Science and Technology, Dalian University of Technology, Dalian 116024, China; School of Computer Science and Technology, Dalian University of Technology, Dalian 116024, China; School of Computer Science and Technology, Dalian University of Technology, Dalian 116024, China; School of Computer Science and Technology, Dalian University of Technology, Dalian 116024, China

## Abstract

**Motivation:**

Significant progress has been achieved in biomedical text mining using deep learning methods, which rely heavily on large amounts of high-quality data annotated by human experts. However, the reality is that obtaining high-quality annotated data is extremely challenging due to data scarcity (e.g. rare or new diseases), data privacy and security concerns, and the high cost of data annotation. Additionally, nearly all researches focus on predicting labels without providing corresponding explanations. Therefore, in this paper, we investigate a more realistic scenario, biomedical few-shot learning, and explore the impact of interpretability on biomedical few-shot learning.

**Results:**

We present LetEx—*Le*arning to *ex*plain—a novel multi-task generative approach that leverages reasoning explanations from large language models (LLMs) to enhance the inductive reasoning ability of few-shot learning. Our approach includes (1) collecting high-quality explanations by devising a suite of complete workflow based on LLMs through CoT prompting and self-training strategies, (2) converting various biomedical NLP tasks into a text-to-text generation task in a unified manner, where collected explanations serve as additional supervision between text-label pairs by multi-task training. Experiments are conducted on three few-shot settings across six biomedical benchmark datasets. The results show that learning to explain improves the performances of diverse biomedical NLP tasks in low-resource scenario, outperforming strong baseline models significantly by up to 6.41%. Notably, the proposed method makes the 220M LetEx perform superior reasoning explanation ability against LLMs.

**Availability and implementation:**

Our source code and data are available at https://github.com/cpmss521/LetEx.

## Graphical Abstract

**Graphical Abstract:**
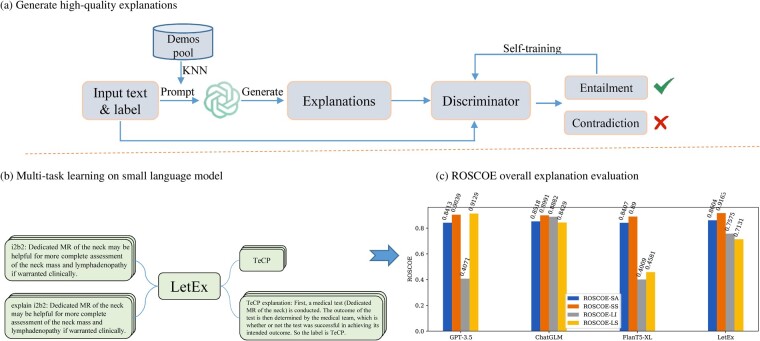

## 1 Introduction

Biomedical literature and electronic medical records are crucial reservoirs of medical knowledge, essential for advancing scientific research and facilitating clinical applications (Guevara *et al.* 2024). However, this medical information typically exists in unstructured or semi-structured natural language formats, posing challenges for physicians to effectively extract useful information from vast amounts of literature and clinical records. Biomedical text mining endeavors to automatically transform unstructured information from biomedical literature into structured knowledge, thereby expediting scientific discovery.

Biomedical text mining, a sub-field of biomedical natural language processing (BioNLP), involves a wide range of subtasks such as biomedical named entity recognition (BioNER), biomedical relation extraction (BioRE), biomedical event extraction (BioEE), and more. Significant research efforts have been dedicated to developing deep learning models in the biomedical field, aiming to assist medical professionals. In recent years, pre-trained language models (PLMs), such as BioBert (Lee *et al.* 2020), PubMedBERT (Gu *et al.* 2022), and ClinicalT5 (Lu *et al.* 2022), have demonstrated superior performance across diverse downstream tasks. These models initially were pre-trained on a large-scale corpus and then finetuned on a specific downstream task in a fully supervised manner. However, the transition of these deep learning models from laboratory to clinical applications presents significant challenges for clinical experts. Current state-of-the-art methods heavily rely on substantial amounts of high-quality data manually annotated by domain experts (Chen *et al.* 2023a). The process of annotating datasets is both time-consuming and difficult, particularly in medical domains, where privacy and security constraints amplify the intricacies of this process. Thus, in real-world applications, the availability of high-quality labeled datasets is usually limited.

Fortunately, aiming at low-resource scenarios, a new paradigm called few-shot learning (FSL) has been proposed (Ma *et al.* 2022). FSL refers to understanding the underlying patterns in the data from only a few training instances to learn new induction and reasoning. Some methods, such as prototype-based and data augmentation-based approaches (Deng *et al.* 2022, Chen *et al.* 2023a), have been proposed for few-shot BioNLP tasks. For example, Deng *et al.* (2022) introduced META-DDIE to predict rare drug–drug interaction events. Aiming to BioNER, Chen *et al.* (2023a) proposed knowledge-guided data augmentation based on semantic relation network. However, almost all existing research focuses on predicting the model’s outcome while neglecting the explainability of the result. Faithful explanations ensure that predicted results are accurate, transparent, and trustworthy (Karim *et al.*, 2023). Understanding how and why model decisions are made can enhance the trust of medical professionals in the system, which is crucial for drug discovery and clinical decisions.

Therefore, in this work, we focus on a more realistic scenario, i.e. biomedical few-shot learning. This setting is realistic, particularly in the clinical domain, where obtaining a few annotations dataset (e.g. 64-shot) is feasible and efficient. Inspired by LLM’s recent outstanding performance in various zero-shot NLP tasks through step-by-step thinking (Brown *et al.* 2020, Wei *et al.* 2022b), we empower this reasoning explanation ability to small-sized models to improve biomedical few-shot learning. Intuitively, reasoning explanations can help the model understand the underlying structure and patterns of the dataset, i.e. how input text is mapped to the label. Thus, we first devised a comprehensive LLM-based workflow using CoT prompting and self-training strategies to collect reasoning explanations corresponding to the labels in the training samples, and used these reasoning chains and labels as supervision signals for the model. Subsequently, we unified all biomedical tasks as text-to-text generation tasks and performed multi-task learning based on the FlanT5 language model to enhance the model’s understanding of the logical mapping between input text and labels, thereby improving its ability to generalize and reason about new samples.

To better validate the effectiveness of the proposed LetEx, this paper investigates from two perspectives: The quality of generated explanations and the performance of models on various few-shot tasks. Experiments are conducted on 3 few-shot settings across 6 biomedical benchmark datasets. The results show that learning from explanation improves the performance of few-shot tasks, outperforming strong baseline models significantly by up to 6.41%. Regarding the quality of reasoning explanations, LetEx demonstrates superior explanation capabilities compared to LLMs across four dimensions: semantic alignment, semantic similarity, logical reasoning, and linguistic coherence. To some extent, our approach can be seen as a small step towards helping biomedical experts make appropriate decisions and interpretable AI. To summarize, our contributions are as follows:

- A suite of complete workflow was designed to obtain high-quality reasoning explanations from LLMs based on CoT prompting and self-training strategies, yielding 32,211 interpretable biomedical samples corresponding to labels across 6 datasets. It is a small step toward interpretable AI in biomedical text mining.
- Compared to the current mainstream methods that only predict labels, our LetEx generates labels and explanations simultaneously. Moreover, the quality of explanations significantly exceeds that of LLMs such as GPT-3.5 and ChatGLM.
- Extensive experiments conducted on three few-shot settings across six biomedical benchmark datasets demonstrate that the proposed LetEx model outperforms strong baseline models by up to 6.41%.

## 2 Related work

*Few-shot learning in BioNLP*. In traditional supervised learning, models are trained on large labeled datasets. However, in practical scenarios, access to large amounts of labeled data can be challenging due to factors such as data scarcity, time, or labor costs. In response, researchers have turned to few-shot learning (FSL) techniques (Yin *et al.* 2020, Agrawal *et al.* 2022, Deng *et al.* 2022, Chen *et al.* 2023a). A core idea of FSL is to enable models to learn induction and generalization only from a few labeled instances. One main line in FSL is the utilization of transfer learning and pre-trained language models (Jia and Zhang 2020, Ge *et al.* 2023). Another avenue of research involves meta-learning approaches. For example, Deng *et al.* (2022) introduces META-DDIE to predict rare drug–drug interaction events. Yin *et al.* (2020) improves the derived prototypes accordingly by prototypical network for few-shot biomedical event trigger identification. Additionally, data augmentation techniques are employed. Aiming to few-shot BioNER, Chen *et al.* (2023a) proposed knowledge-guided data augmentation based on semantic relation network. Recently, LLMs have brought further performance improvements on few-shot clinical information extractors (Agrawal *et al.* 2022, Oniani *et al.* 2022). Unlike the above methods, we handle all of BioNLP tasks in a unified manner.

*Learning from explanations*. Explanations can help biomedical experts to better understand the behaviors of deep learning models, which are pivotal in practical applications such as medical decisions, drug discovery, and predicting diseases. Previous studies have demonstrated satisfactory performance (Gonzalez-Agirre *et al.* 2019, Lee *et al.* 2020, Chen *et al.* 2023b, Zhou *et al.* 2024), but cannot provide faithful explanations of the predicted results. Recently, learning with explanations has received increasing attention. For example, Wei *et al.* (2022a) used few-shot CoT prompting to elicit GPT-3 to generate answers step by step. Yu *et al.* (2022) fine-tuned reasoning chains to enhance the reasoning ability of language models. In the biomedical domain, Parmar *et al.* (2022) proposed multi-task instruction tuning integrating positive examples with their explanations to enhance FSL. Different from these works, we first focus on reasoning explanations of small-sized language models. Secondly, we provide answers and explanations at the same time, rather than using reasoning explanations before predicting answers.

## 3 Materials and methods

### 3.1 Explanation generation from LLM with self-training

**Figure 1.**
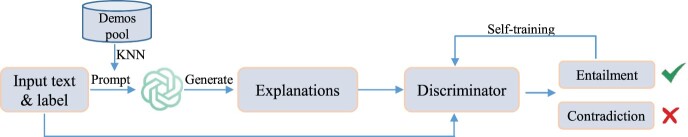
An overview of faithful explanation generation with CoT prompt and self-training strategy. Finally, entailment (i.e. correct) reasoning explanations are retained, and contradiction (i.e. false) reasoning explanations are discarded.

A main challenge is that the current biomedical datasets lack annotation explanations of labels. To this end, we first collect the reasoning explanations corresponding to each sample label from the training set using an LLM, without accessing the model parameters. Figure 1 illustrates a suite of complete explanation generation workflow, designed using LLM-based CoT prompting and self-training strategies. Concretely, it contains three consecutive stages: CoT prompt generation, discrimination and filtering, and self-training.

*CoT prompt generation*. To generate high-quality explanations, we utilize CoT prompting (Wei *et al.* 2022a) to elicit LLMs to generate reasoning explanations step by step. Specifically, a demonstration pool is initially constructed. We randomly select a few (e.g. 20) examples ($x_{i}^{d}$, $y_{i}^{d}$) from each training dataset, and manually craft a reasoning explanation $r_{i}^{d}$ for each instance according to the annotation guidelines, thereby constituting the candidate demonstration pool ${\left\{P_{i}=(x_{i}^{d},r_{i}^{d},y_{i}^{d})\right\}}_{i=1}^{20}$. Then, given the training sample $x_{i}$, we use *k*-nearest neighbors (KNN) to dynamically select one demonstration $P_{i}$ from candidate demostrations pool that most closely resembles $x_{i}$. Next, we append each demonstration $P_{i}$ to the training sample pair $x_{i}$ and $y_{i}$, using it as input to prompt the LLM to generate a reasoning explanation $r_{i}$, i.e.

(1) $$r_{i}∼\mathrm{LLM}\left(r_{i}|P_{i},x_{i},y_{i}\right)$$

**Figure 2.**
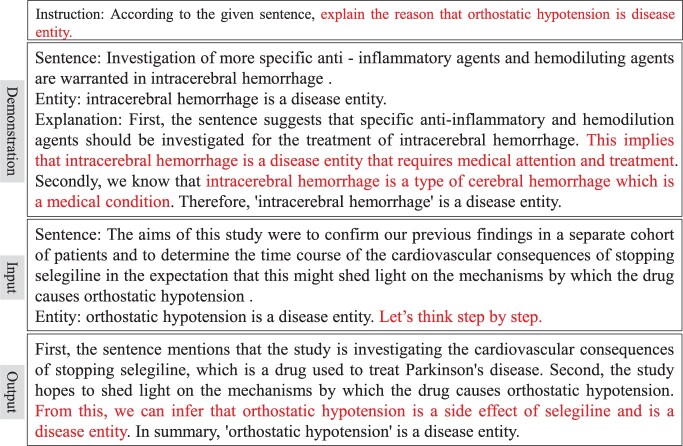
An example of CoT prompt in the BioNER task employs a demonstration with label and reasoning explanation to elicit the LLM to generate a target explanation.

An example of CoT prompting is presented in Fig. 2. In contrast to Wei *et al.* (2022a), we leverage the input text $x_{i}$ and label $y_{i}$ to generate reasoning explanations $r_{i}$, aiming to minimize potential errors in the explanation.

*Discrimination and filtering*. However, the hallucination of LLMs is still inevitable. Prior work conducts a human evaluation (Marasovic *et al.* 2022) to determine the quality of reasoning. Generally, a high-quality reasoning explanation implicitly includes propositional logic related to the input text. In other words, when there is an entailment relationship between the input text and the reasoning explanation, the generated explanation is considered reliable. This is similar to a text entailment task. Thus, the problem is converted into a text entailment task by mapping ($x_{i}$, $r_{i}$) into ($p_{i}$, $h_{i}$), where $x_{i}$ and $r_{i}$ are viewed as premise $p_{i}$, and hypothesis $h_{i}$, respectively, and the label is assigned as either entailment or contradiction. Technically, we train a nature language inference (NLI) discriminator to filter noise samples ($x_{i}$, $r_{i}$), where explanations $r_{i}$ are prone to hallucination. Specifically, we adopt pretrained PubMedBERT-MNLI-MedNLI (Deka *et al.* 2023), trained on the MultiNLI (Williams *et al.* 2018), and MedNLI (Romanov and Shivade 2018) datasets, to serve as NLI discriminators by further fine-tuning. The discriminator is also a few-shot learning. By comparing the final predicted label of the explanation $r_{i}$ with the ground-truth label $y_{i}$ of the input text $x_{i}$, we identify evident error text-explanation pairs (i.e. premise-hypothesis pairs) and assign their label as a contradiction. Similarly, previously manually crafted explanations for each dataset are considered as entailment labels. Based on the converted premise-hypothesis samples, we further fine-tune the NLI discriminator.

*Self-training*. Then we adopt a self-training strategy to improve discriminator. Concretely, the predictions for the entailment label with the highest confidence are collected as new training samples, which are then used to re-train an improved discriminator model. After two to three iterations, we ultimately filtered out 1100 error explanations samples and retained 32 211 high-quality interpretable training samples across six datasets. These retained samples are used to construct support sets for simulating few-shot scenarios in Section 4.2.

### 3.2 Learning from explanation based on multi-task

**Figure 3.**
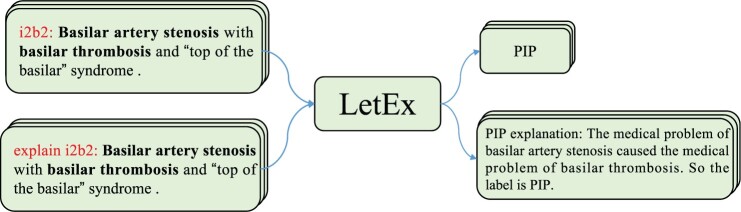
An overview of the proposed LetEx model for biomedical few-shot learning. On the i2b2 2010 dataset, given an input sentence containing the entity pairs ‘Basilar artery stenosis’ and ‘basilar thrombosis’ under different task prefixes (i2b2 represents relation extraction task and explan i2b2 denotes label explanation generation task), LetEx simultaneously predicts the relation label PIP and generates the corresponding explanation. The PIP label identifies medical problems that describe other aspects of the same issue or cause other medical problems.

Intuitively, reasoning explanations provide detailed information on how target label $y_{i}$ can be derived from the input text $x_{i}$, usually containing task-related knowledge that is difficult to obtain solely from the text $x_{i}$. In other words, these reasoning explanations offer additional supervision for training samples, enhancing the understanding of the logical mapping between text-label pairs. Hence, we propose the LetEx model, which learns from explanations as illustrated in Fig. 3. The LetEx model takes an input sentence with task-specific prefixes and simultaneously generates both labels and their corresponding explanations. The backbone of the model is based on the 220M parameter FlanT5-base. Different prefixes represent different tasks. The label prediction task uses [task_prefix: ] and the explanation generation task utilizes [explain task_prefix: ] as the model prefix, where task_prefix usually is the name of the dataset. In the case of BioRE (i2b2 2010 dataset), the model receives the text sequence ‘Basilar artery stenosis with ⋯ syndrome.’ and then is trained to generate the target label ‘PIP’ along with the corresponding reasoning explanation ‘PIP explanation: The medical problem of ⋯ So the label is PIP.’. The prefixes ‘i2b2: ’ and ‘explain i2b2: ’ indicate the relation extraction task and the explanation generation task, respectively. Learning to explain enables the model to better generalize the target labels rather than overly relying on memorizing the current input text. The training objective of LetEx model is to joint loss $L_{\mathrm{label}}$ and $L_{\mathrm{expl}}$:

(2) $$L_{\text{label}}=-\frac{1}{N}\sum_{i=1}^{N}y_{i}\;\text{log}({\hat{y}}_{i})$$

(3) $$L_{\text{expl}}=-\frac{1}{N}\sum_{i=1}^{N}r_{i}\;\text{log}({\hat{r}}_{i})$$

(4) $$L=\lambda L_{\text{label}}+(1-\lambda )L_{\text{expl}}$$

where *N* represents the number of training samples, $y_{i}$ (or $r_{i}$) is the ground truth label (or explanation) for the *i*th sample, and ${\hat{y}}_{i}$ (or ${\hat{r}}_{i}$) is the corresponding prediction. $L_{\text{label}}$ (or $L_{\text{expl}}$) represents the cross-entropy loss for the generated label (or explanation) prediction, λ is a hyper-parameters for the loss of label and explanation.

## 4 Experiments

### 4.1 Datasets and evaluation metrics

The LetEx model is evaluated on 6 popular benchmark datasets involving three biomedical tasks.

1. *BioNER*. NCBI (Dogan *et al.* 2014) and BC5CDR-disease (Li *et al.* 2015) are utilized to extract disease entities in this task. To identify the named entities of the biomedical text, we insert the corresponding entity labels around the entity spans as target output during the training phase, e.g. for the input text ‘Genotype and phenotype in patients with dihydropyrimidine dehydrogenase deficiency’, the target output is ‘Genotype and phenotype in patients with disease* dihydropyrimidine dehydrogenase deficiency *disease’.
2. *BioRE*. The task of BioRE aims to extract the relation of entities. We conduct relation extract on the i2b2 2010 (Uzuner *et al.* 2011), HPRD50 (Fundel *et al.* 2007), and AIMed (Bunescu *et al.* 2005). The former originates from clinical medical notes, whereas the latter two come from biomedical literature. For this task, we directly generate the corresponding label text.
3. *NLI*. MedNLI (Romanov and Shivade 2018) dataset is employed for NLI task. The MedNLI consists of sentence pairs with premise and hypothesis, where the task is to determine the logical relationship between them, typically categorized as ‘entailment’, ‘contradiction’, or ‘neutral’. We packet the premise-hypothesis pair to a text sequence. More details about datasets can be found in Supplementary Appendix A.

In our experiments, to evaluate the few-shot performance across three distinct tasks, micro-averaged F1 scores are utilized for entity and relation extraction, while accuracy is used for the NLI task. More details on the experimental implementation are provided in Supplementary Appendix B.

### 4.2 Low-resource setting

In the few-shot setting, we follow *N*-way *K*-shot setting (Ma *et al.* 2022, Chen *et al.* 2023a), where *N* classes are randomly sampled from the retained training samples, with *K* examples per class, to construct a support set $S=\left\{(x_{i},y_{i},r_{i}){|}_{i=1}^{N\times K}\right\}$ for training. In here, $x_{i}$ and $y_{i}$ denote input text and corresponding gold label, $r_{i}$ is reasoning explanation related to lable $y_{i}$, and all categories in the dataset are considered, i.e. N=|C|. Then, the model is evaluated on all test (query) samples *Q* (S∩Q=∅). Although N*K samples are chosen for the support set *S*, the total number in *S* could exceed N*K (such as in BioNER task). Therefore, the same downsampling algorithm is adopted as by Hou *et al.* (2020) to construct support set. In the work, considering practical scenarios, we simulated three low-resource scenarios, namely 16-shot, 32-shot, and 64-shot.

### 4.3 Compared methods

The primary goal of our study is to train a small-size model that can match or outperform LLMs in the performance of both domain-special tasks and generated explanations. In this way, in practical applications, we can avoid high computational costs when deploying LLMs. To measure this goal, the large language models by zero-shot CoT prompt and domain-special small-sized language models based on vanilla fine-tuning are considered as the baseline models, specifically (1). LLMs, ranked by their scale, include the 175B GPT-3.5-turbo, 6B ChatGLM (Du *et al.* 2022), and 3B FlanT5-XL (Chung *et al.* 2022), all of which exhibit robust CoT reasoning capabilities. Following (Kojima *et al.* 2022), ‘Let’s think step by step’ is appended to the prompt instructions to predict target tasks with LLMs (detailed prompt instructions are given in Supplementary Appendix C). (2). Small-sized language models include encoder-only domain-special PLMs such as BioBERT (Lee *et al.* 2020) and PuMedBERT (Gu *et al.* 2022), and encoder–decoder PLMs like T5 (Raffel *et al.* 2020) and ClinicalT5 (Lu *et al.* 2022).

## 5 Main results

Table 1 presents the experimental results in few-shot scenarios, specifically 16-shot, 32-shot, and 64-shot, across six biomedical benchmark datasets. Overall, the proposed LetEx demonstrates superior performance across three few-shot scenarios in all datasets. Firstly, LetEx demonstrates superior performance compared to current LLMs across six datasets in the 16-shot setting, significantly surpassing GPT-3.5-turbo with 175 billion parameters. Notably, LetEx achieves F1 score improvements of up to 17.15% on the HPRD50 dataset and 17.2% on the AIMed dataset. The same increasing trend is observed with LLMs having either 3B FlanT5-XL or 6B ChatGLM. Secondly, compared to T5 and ClinicalT5, LetEx with the same parameter scale achieves a 2.14% improvement in F1 score on the i2b2 2010 dataset, a 4.29% improvement in F1 score on the HPRD50 dataset, and a 6.41% increase in accuracy on the MedNLI dataset, under a 32-shot setting. These observations suggest that learning from explanations helps LetEx gain a deeper understanding of the underlying mappings between text-label pairs, enhancing its comprehension of the data rather than merely memorizing labels of training samples. This approach allows the model to capture complex patterns and intrinsic structures of the data, ultimately improving its generalization and reasoning ability when handling new samples. A possible reason for the significant improvement in MedNLI is that the base model of LetEx, FlanT5-base, has undergone specialized instruction fine-tuning on other textual entailment datasets, which has enhanced LetEx’s generalization and reasoning capabilities for similar tasks. Additionally, LetEx provides corresponding explanations for the prediction results, which is crucial in clinical scenarios. This feature is not available in the same scale language models, such as T5, ClinicalT5, BioBERT, or PubMedBERT. We also find that although LLMs perform well in various tasks, fine-tuning task-specific small-size models can make their performance on target tasks still match or even surpass those LLMs. For example, under 16-shot setting, the vanilla fine-tuning based on encoder–decoder PLMs achieved on NCBI and BC5CDR-disease comes close to 175B GPT3.5-turbo.

**Table 1.** Performance comparison with SoTA methods across all datasets.^a^

Model	Params.	Expl.	NCBI			BC5CDR-disease			MedNLI		
			16-shot	32-shot	64-shot	16-shot	32-shot	64-shot	16-shot	32-shot	64-shot
GPT-3.5-turbo^▲^	175B	✓	50.49^†^			51.77^†^			63.71		
ChatGLM^▲^	6B	✓	30.33			23.70			64.13		
FlanT5-XL^▲^	3B	✓	30.86			29.21			47.12		
BioBERT	110M	✗	43.01	51.83	69.32	37.99	47.63	60.03	49.64	60.54	65.33
PubMedBERT	110M	✗	43.44	54.39	67.70	43.09	50.19	64.57	49.08	57.10	67.29
T5	220M	✗	48.96	60.80	69.08	51.09	**59.55**	66.84	67.93	64.76	70.04
ClinicalT5	220M	✗	49.97	63.49	69.83	44.27	59.53	**66.90**	51.89	64.90	67.79
LetEx(our)	220M	✓	**50.94**	**63.50**	**70.28**	**53.67**	58.89	65.17	**70.53**	**71.31**	**73.00**

Model	Params.	Expl.	i2b2 2010			HPRD50			AIMed		
			16-shot	32-shot	64-shot	16-shot	32-shot	64-shot	16-shot	32-shot	64-shot

GPT-3.5-turbo^▲^	175B	✓	50.49			47.14			48.19		
ChatGLM^▲^	6B	✓	36.73			57.14			67.50		
FlanT5-XL^▲^	3B	✓	14.31			50.00			67.30		
BioBERT	110M	✗	61.38	71.30	76.79	54.29	58.57	62.86	47.21	55.07	51.05
PubMedBERT	110M	✗	55.27	74.45	80.39	55.71	54.29	68.57	43.01	62.65	65.11
T5	220M	✗	**64.18**	74.71	80.89	**68.57**	70.00	80.00	63.83	**65.11**	**69.49**
ClinicalT5	220M	✗	64.16	75.63	79.33	60.00	62.85	77.14	63.65	63.37	68.40
LetEx(our)	220M	✓	63.70	**77.77**	**82.53**	64.29	**74.29**	**84.29**	**65.39**	57.17	68.86

a ‘Params.’ denotes the model’s parameter size, and ‘Expl.’ indicates whether the model has an explainable ability. The best at the same scale is bolded.

† indicates results are reported by (Chen *et al.*, 2023c).

▲ denotes results obtained by zero-shot CoT.

## 6 Discussion

### 6.1 Effect of learning from explanations

**Figure 4.**
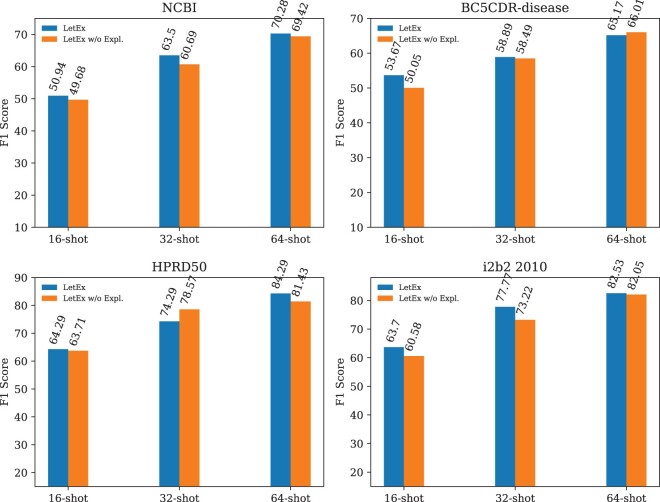
Performance comparison of LetEx trained with and without explanations on various datasets under different few-shot scenarios.

A series of ablation studies are conducted to validate the effect of learning from explanations, as shown in Fig. 4. It can be concluded that learning from explanations makes LetEx perform better. Refocusing the LetEx model solely on label predictions, without corresponding explanations, resulted in varying degrees of reduction in F1 scores. Except for HPRD50, reductions of 2.81%, 0.5%, and 4.55% were observed on NCBI, BC5CDR-disease, and i2b2 2010 dataset, respectively, under the 32-shot setting. The same declining trend can be observed in both the 16-shot and 64-shot settings. It demonstrates that most of the benefits come from the explanations. The main reason is that learning from explanations allows the model to induce and generalize training samples by understanding and reasoning the connection between input text and label pairs.

### 6.2 The gap with full supervision

**Figure 5.**
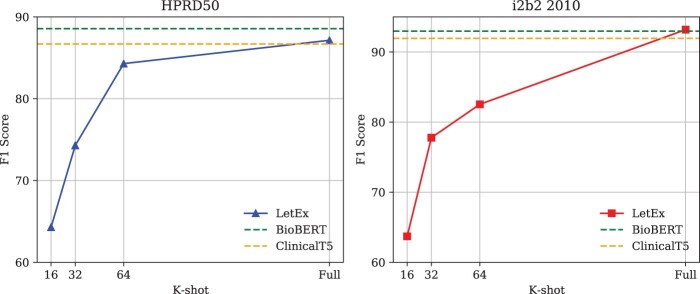
The gap between few-shot learning and full supervision. On two datasets, LetEx achieves comparable performance against standard fine-tuning on the full dataset, even using much fewer training samples (e.g. 64-shot on the HPRD50 dataset).

We further investigate the gap between few-shot learning and full supervision. Figure 5 illustrates the performance variation of LetEx from *k* = 16 to full supervision on the HPRD50 and i2b2 2010 datasets. Compared to the baseline model fine-tuned with the ClinicalT5 on the full dataset, LetEx achieves comparable performance on HPRD50 with only 64-shot, resulting in an 11-fold increase in data efficiency. Similar observation also appeared on i2b2 2010 dataset. As the training samples increase from low resources to the full dataset, the gap between the two continues to narrow. Meanwhile, LetEx also shows decent performance on both full datasets. In comparison to fine-tuning ClinicalT5, the proposed LetEx model outperforms 0.46% and 1.25% F1 score on HPRD50 and i2b2 2010, respectively. While falling behind by 1.42% compared to fine-tuned BioBert on the HPRD50 dataset, LetEx maintains a slight advantage on the i2b2 dataset.

### 6.3 Quality of generated explanation

**Figure 6.**
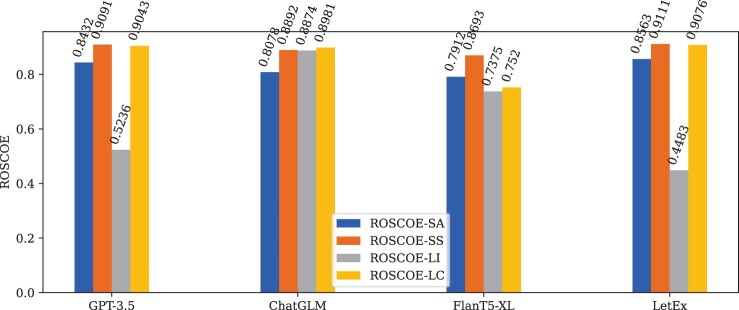
ROSCOE overall explanation evaluation results on the HPRD50 dataset across from 175B GPT-3.5 to 220M LetEx.

To objectively evaluate the quality of generated explanations, we utilize the ROSCOE (Golovneva *et al.* 2023), a suite of scoring metrics for reasoning chains, to measure the correlation between text-label pair and generated explanations across four dimensions, i.e. semantic alignment (ROSCOE-SA), semantic similarity (ROSCOE-SS), logical inference (ROSCOE-LI), and language coherence (ROSCOE-LC). Each metric is constrained within the range of [0, 1], where 1 signifies highest correlation, while 0 means irrelevant. We include more ROSCOE details in Supplementary Appendix D. We report the quality evaluation of the explanations generated from the HPRD50 test datasets, which are summarized in Table 2 and Fig. 6. Overall, compared to baseline models, LetEx performs better across most metrics after being trained on high-quality explainable datasets. We observe that 3B FlanT5-XL usually directly generates answers and does not provide corresponding reasoning explanations, resulting in lower semantic overlap with the input text. Consequently, the generated explanations have lower correlations on Faithfulness-*, Info-Step, Perplexity-Chain, and Perplexity-Step. Notably, the Repetition-* and Self-Consistency measure the correlations between reasoning steps in explanations. Therefore, there are no Repetition-* and self-consistency scores as there are no reasoning steps to compare. As scaling up model parameters, this trend diminishes gradually from 6B ChatGLM to 175B GPT-3.5-turbo, bringing higher quality explanations. Rather than pursuing unlimited expansion of model parameters, the quality of explanations generated by 220M LetEx models, trained to specific tasks, is higher, which enhances confidence in clinical medical decision-making and facilitates faster deployment model in practice. Additionally, we assess the explanation quality of the i2b2 2020 dataset, the detailed analysis results are presented in Supplementary Appendix D.

**Table 2.** Evaluate the explanation ability of the HPRD50 dataset using all ROSCOE metrics.^a^

	175B GPT-3.5-turbo	6B ChatGLM	3B FlanT5-XL	220M LetEx
ROSCOE-SA				
Faithfulness-Step	0.7684	0.7569	0.7385	**0.8290**
Faithfulness-Token	0.8432	0.8078	0.7912	**0.8547**
Info-Step	0.8045	0.7671	0.7385	**0.8563**
Repetition-Token	0.0945	**0.7377**	n/a	0.1144
ROSCOE-SS				
Info-Chain	0.9091	0.8892	0.8693	**0.9111**
Repetition-Step	0.0458	**0.7181**	n/a	0.0589
ROSCOE-LI				
Source Consistency	0.5236	0.5881	**0.7375**	0.4483
Self-Consistency	0.3954	**0.8874**	n/a	0.2996
ROSCOE-LC				
Perplexity-Step	**0.0098**	0.0072	0.0001	0.0084
Perplexity-Chain	**0.0748**	0.0204	0.0001	0.0516
Grammar	0.9043	0.8981	0.7520	**0.9076**

a The highest correlation is bolded and the second best is underlined in each row.

### 6.4 Impact of explanation quality on few-shot learning

The success of LetEx relies on the quality of the explanations collected from LLMs during the training phase. Therefore, we further explore how the quality of the collected generative explanations during training affects the performance of the LetEx model.

Specifically, based on the complete workflow on explanation generation in Fig. 1, we conduct ablation study on AIMed and MedNLI datasets by removing different settings. As shown in Table 3, the test results for the AIMed and MedNLI datasets across various few-shot scenarios are reported using the F1 score and accuracy, respectively. We first removed only the Discriminator, leading to the most significant performance drop among all factors, ranging from 0.35 to 3.11. This indicates that the Discriminator plays a crucial role in filtering out erroneous explanation samples and retaining high-quality, interpretable training samples. When demos are ablated, i.e. without demonstration $P_{i}$ in Equation (1), the performance of LetEx shows slight fluctuations across various few-shot settings. This is primarily because the GPT-3.5-turbo language model is sufficiently powerful to generate reasonable explanations for training sample labels even in zero-shot scenarios. When labels are removed, we observe a consistent decline in various few-shot scenarios. Additionally, when we replace GPT-3.5-turbo with the ChatGLM language model-to-generate label explanations for the training samples, the performance declines to varying extents, with reductions of up to 2.75 F1 score.

**Table 3.** Impact of collected explanation quality during training on LetEx.^a^

Model	AIMed			MedNLI		
	16 shot	32 shot	64 shot	16 shot	32 shot	64 shot
LetEX	**65.39**	57.17	**68.86**	**70.53**	**71.31**	**73.00**
w/o Discriminator	62.28	56.43	66.48	70.18	70.74	72.50
w/o demos	63.28	**58.35**	68.67	70.46	70.95	72.92
w/o label	65.11	54.70	67.48	70.04	71.09	72.78
LLM*	64.56	54.42	68.03	70.32	70.81	72.85

a LLM* means replacing GPT-3.5-turbo with a ChatGLM to generate label explanation.

The best score at the same few-shot scenario is bolded.

Overall, all these settings ensure that the label explanations collected during training are high quality. High-quality explanations provide more accurate logical mapping between text-label pairs, which can significantly improve the performance of the LetEx model in few-shot learning. Conversely, poor-quality explanations may introduce ambiguity or misinformation, leading to suboptimal performance. Subsequently, we explored how the explanations collected under different settings during the training phase impact the quality of explanations generated by LetEx during the testing phase. More details are included in Supplementary Appendix E.

### 6.5 Case study

In order to evaluate the advantages of the proposed LetEx method, we conduct further analysis by random sampling on the i2b2 2010 dataset, as shown in Table 4. We find that FlanT5-XL and ChatGLM directly predict the answers without giving reasoning steps in case 1. Worse still, ChatGLM produces the wrong answer. Although GPT-3.5 provided corresponding reasoning explanations, the incorrect reasoning led to an ultimately wrong prediction result. In contrast, LetEx pointed out that the sentence did not mention the outcome of the treatment, and thus the relation labels is TrAP. A similar situation occurred in Case 2. Both FlanT5-XL and ChatGLM directly generated relation labels. Notably, ChatGLM repeated the answer during the explanation without providing any reasoning steps. Compared to GPT-3.5, ChatGLM, and FlanT5-XL, our LetEx generates correct answers and faithful explanations in both cases. By learning from the explanation, our LetEx gains the ability that understand the underlying structure and patterns of the dataset, i.e. how the input texts are mapped to pre-defined labels. Therefore, LetEx not only accurately predicts labels but also generates reliable explanations in low-resource settings. More case studies can be found in Supplementary Appendix F.

**Table 4.** Case study of explanations and predicted labels generated by LetEx and other LLMs on the i2b2 2010 dataset.^a^

Case 1: **Text**: The patient is a 40-year old woman with end-stage renal disease, on hemodialysis since 12/91. **Relation:** TrAP	

175B GPT-3.5	**Explanation:** 1. Identify the entities: ‘hemodialysis’ and ‘end-stage renal disease’ 2. Determine the relationship between the entities: The sentence states that the patient is undergoing hemodialysis due to end stage renal disease, indicating a ‘TrIP’ (treatment improves or cures medical problem) relationship. 3. Final answer: TrIP.
	**Relation:** TrIP
6B ChatGLM	**Explanation:** –
	**Relation:** False
3B FlanT5-XL	**Explanation:**: –
	**Relation:** TrAP
220M LetEx	**Explanation:** The sentence mentions that the patient has endstage renal disease. It also states that the patient is on hemodialysis since 12/91. However, it does not mention the outcome of the treatment, whether it was successful or not. So the label is TrAP.
	**Relation:** TrAP

Case 2: **Text**: Chest X-ray showed a question of left lung opacity. **Relation:** TeRP	

175B GPT-3.5	**Explanation:** The sentence mentions a test, specifically a chest X-ray, and the outcome of the test is known, which is that it showed a ‘question of left lung opacity’. Therefore, the relation between the head entity ‘chest X-ray’ and tail entity ‘left lung opacity’ is ‘TeRP’ (test reveals medical problem).
	**Relation:** TeRP
6B ChatGLM	**Explanation:** Relation_type: False
	**Relation:** False
3B FlanT5-XL	**Explanation:** –
	**Relation:** TrAP
220M LetEx	**Explanation:** First, a chest X-ray was conducted on the patient. Then, the outcome of the test revealed the presence of a question of left lung opacity. So the label is TeRP.
	**Relation:** TeRP

a – signifies no generating explanation.

### 6.6 Limitations of proposed model

As mentioned in Section 6.4, the success of the LetEx relies on the quality of the explanations collected from LLMs. Although we show great improvement on the six biomedical benchmark datasets, if the task involves complex knowledge reasoning, the explanation generated by LLMs will become less credible. Additionally, during filtering, NLI discriminators can only approximate human judgments regarding the plausibility of generated explanations, inevitably introducing additional noise. Combining biomedical domain knowledge with LLMs to generate robust reasoning explanations will be an interesting direction for future work.

## 7 Conclusion

In this article, considering realistic scenarios, we propose a novel LetEx method that learns from the explanations to enhance the inductive reasoning ability of few-shot learning. Specifically, we initially gathered high-quality explanations by devising a suite of complete workflow based on LLMs through CoT prompting and self-training strategies. Then we convert all biomedical NLP tasks into a text-to-text generation task in a unified manner. These reasoning chains provide additional supervision for LetEx through multi-task training and prompt learning, enabling it to predict labels and corresponding explanations simultaneously. Experiments are conducted on 3 few-shot settings across six biomedical benchmark datasets and the results show that learning from explanation could improve the performance of few-shot tasks, outperforming strong baseline models significantly by up to 6.41%. In addition, the quality of generated explanations from LetEx is superior obviously to LLMs such as GPT-3.5-turbo and ChatGLM, which enhances confidence in clinical medical decision-making and facilitates faster deployment models in practice.

## Supplementary Material

btae589_Supplementary_Data

## Data Availability

The LetEx data underlying this article are available at https://github.com/cpmss521/LetEx.
