# Supplementary material for: Learning to explain is a good biomedical few-shot learner
Source: Bioinformatics. 2024 Oct 3;40(10):btae589. doi: 10.1093/bioinformatics/btae589 (PMC11483110; doi:10.1093/bioinformatics/btae589)
Supplement: btae589_Supplementary_Data [file btae589_supplementary_data.pdf]

Appendix

A Datasets

We conducted experiments using six biomedical benchmark datasets across three biomedical text mining tasks. The statistics for these datasets are provided in Table 5, and detailed descriptions of each dataset are as follows:

- NCBI: The NCBI dataset is a biomedical corpus (Dogan *et al.*, 2014) containing 793 PubMed abstracts, each manually annotated to include disease mentions and their corresponding concepts, providing a high-quality gold standard for disease name recognition and normalization research.
- BC5CDR-disease: BioCreative V Chemical-Disease Relation task (BC5CDR) (Li *et al.*, 2015) is annotated for biomedical named entity recognition and relation extraction, consisting of 1500 PubMed articles, covering annotations of disease and chemical entities, as well as their interactions. In this paper, we only consider the disease entity of the named entity recognition task.
- i2b2 2010: The i2b2 2010 (Uzuner *et al.*, 2011) dataset was sourced from three distinct medical institutions and was annotated by medical professionals to identify eight types of relations between medical problems and corresponding treatments, i.e., TrIP, TrWP, TrCP, TrAP, TrNAP, PIP, TeRP, TeCP.
- HPRD50: The HPRD50 (Fundel *et al.*, 2007) dataset is sourced from the HPRD database and used for studying human protein-protein interactions (PPI). HPRD50 corpus consists of 43 documents annotated by true and false protein-protein interaction (PPI) relation.
- AIMed: The AImed (Bunescu *et al.*, 2005) dataset is developed to evaluate protein name recognition and protein-protein interaction (PPI) extraction. AIMed corpus consists of 225 documents annotated by true and false protein-protein interaction (PPI) relation.
- MedNLI: The MedNLI (Romanov and Shivade, 2018) is collected from MIMIC-III with a form of premise-hypothesis pairs. And annotated by radiologists, the dataset is graded for entailment, contradiction, or neutrality based on whether the premise entails the hypothesis.

B Implementation details

Due to privacy considerations, we employ GPT-3.5-turbo with the Azure OpenAI API to collect explanations using greedy decoding and a temperature setting of 0.7. The same prompt instructions are given to all LLMs for each task. We fine-tune small-sized language models using a learning rate of 5e-5 and a batch size of 8, with a maximum sentence truncation length set to 256. And we set the loss weight λ to 0.6 during training. The experiments were conducted using the Huggingface library (Wolf *et al.*, 2020) on two NVIDIA RTX 4090 GPUs.

C Prompt Instruction

CoT prompts are listed from Table 6 to Table 9 for different datasets. We provide detailed descriptions for each relation label on the BioRE task. Following (Sanyal *et al.*, 2022), the prompt instruction in MedNLI is used as follows: "*premise*" Based on the previous passage, is it entail "*hypothesis*"?.

D Evaluation of reasoning explanation

Unlike traditional evaluation metrics, ROSCOE (Golovneva *et al.*, 2023) is specifically designed to assess the quality of reasoning explanations generated by LLMs. It focuses on four dimensions: semantic alignment (ROSCOE-SA), semantic similarity (ROSCOE-SS), logical inference (ROSCOE-LI), and language coherence (ROSCOE-LC). ROSCOE-SA is designed to evaluate whether the reasoning produced by a model aligns with the provided context. It calculates the normalized cosine similarity between each hypothesized reasoning step and the most similar sentences in the source text, generating a reasoning alignment vector. This vector quantifies how well the step-by-step reasoning is grounded in the source text. ROSCOE-SS measures semantic equivalence between different texts by evaluating them as a whole, rather than focusing on individual text units as ROSCOE-SA does. ROSCOE-LI is used to measure logical errors between pieces of text. ROSCOE-LC involves both the fluency and grammatical correctness of the text, primarily measured through perplexity and grammatical acceptability. This enables a more comprehensive evaluation of the model's reasoning explanation ability. For more details, it is recommended to consult the original paper (Golovneva *et al.*, 2023).

The reasoning quality evaluation report of the i2b2 2010 test dataset can be found in Figure 7 (overall metrics) and Table 10 (detail metrics). We can see that the 220M LetEx model produces high ROSCOE-SA and ROSCOE-SS compared to LLMs like GPT-3.5, ChatGLM and FlanT5-XL, which means that generated explanation of our model is more faithful to the source text. We also found that FlanT5-XL nearly does not produce reasoning chains, leading to terrible scores in each aspect. It may be that FlanT5-XL does not adapt well to the prompt.

E Impact of Training Explanations on LetEx Explanation Quality

In this part, we explored how the explanations collected under different settings during the training phase impact the quality of explanations generated by LetEx during the testing phase. Ablation studies are conducted on the AIMed dataset with a 16-shot scenario, removing different components from Figure 1. The label explanation quality during the test phase is shown in Figure 8. We observed that removing different settings results in variations in the quality of label explanations collected during the training phase, which in turn affects the label explanations generated during the testing phase. Notably, when the discriminator is removed, the ROSCOE-SA metric drops significantly, indicating that the generated label explanations do not align well with the context, leading to a lower ROSCOE-SA score. Removing demonstrations and labels also impacts the quality of generated label explanations in the testing phase. Additionally, replacing the GPT-3.5-turbo language model with ChatGLM introduces more logical errors and reduces text fluency in the generated label explanations compared to LetEx.

F Examples of Case Studies

We also randomly selected some samples from the HPRD50 test dataset and reported the reasoning explanations of different language models. As shown in Table 11, the GPT-3.5-turbo model tends to produce long chains, while ChatGLM generates shorter chains. In contrast, FlanT5-XL often generates direct answers. However, LetEx, trained on high-quality explanations, consistently generates more faithful explanations and accurate predictions.

References

R. C. Bunescu et al. (2005). Comparative experiments on learning information extractors for proteins and their interactions. *Artif. Intell. Medicine*, **33**(2), 139–155.

R. I. Dogan et al. (2014). NCBI disease corpus: A resource for disease name recognition and concept normalization. *J. Biomed. Informatics*, **47**, 1–10.

K. Fundel et al. (2007). Relex - relation extraction using dependency parse trees. *Bioinform.*, **23**(3), 365–371.

Table 5. Dataset statistics used in biomedical few-shot learning. Where "–" indicates that the data is officially unavailable.

Dataset	Support Set			Dev	Test
	16 shot	32 shot	64 shot		
NCBI	16	32	64	923	940
BC5CDR-disease	16	32	64	4581	4797
MedNLI	48	96	192	1395	1422
i2b2 2010	144	279	516	-	13381
HPRD50	32	64	128	-	70
AIMed	32	64	128	-	1095

Table 6. CoT prompt for NCBI and BC5CDR-disease

Considering "disease" type of named entities, recognize all disease entities in the given sentence.
Given the final answer with the format of ["entity_type", "entity_name"]. If no entity exists, then answer [ ]
sentence: The APC gene , responsible for familial adenomatous polyposis , is mutated in human gastric cancer .
Let's think step by step.

Table 7. CoT prompt for i2b2 2010

Consider predefined relation types {"TrIP": "treatment improves or cures medical problem", "TrWP": "treatment worsens medical problem, including treatment does not improve or cure the problem", "TrCP": "treatment causes medical problem", "TrAP": "treatment is administered for medical problem, and the treatment outcome is not mentioned", "TrNAP": "Treatment is not administered because of medical problem", "PIP": "medical problems cause other aspects of the same medical problem or cause other medical problems", "TeRP": "test reveals medical problem, including where a test is conducted and the outcome is known", "TeCP": "test conducted to investigate medical problem, including where a test is conducted and the outcome is not known", "False": "There is no relation between entity pairs"}, classify relation between "percocet" and "allergic" pairs from the given sentence and give the final answer with the format of ["relation_type"].
sentence: The patient is allergic to Hydralazine , codeine , ace inhibitors , Nifedipine and Percocet .
Let's think step by step.

Table 8. CoT prompt for HPRD50 and AIMed

Consider predefined relation types {"PPI": "protein-protein interactions", "False": "no evident interaction between protein entity pairs"}, classify relation between "@PROTEIN1$" and "@PROTEIN2$" pairs from the given sentence and give the final answer with the format of ["relation_type"].
sentence: @PROTEIN1$ promotes coprecipitation of p85 with gp130, the signal-transducing component of the @PROTEIN2$
Let's think step by step.

Table 9. CoT prompt for MedNLI

"In [**8-7**] had echo which showed diastlolic relaxation dysfunction, hyperdynamic LV, and LVOT gradient induced to 50 mmHg." Based on the previous passage, is it entailment "History of hypertension" ? entailment, contradiction, or neutral?
Let's think step by step.

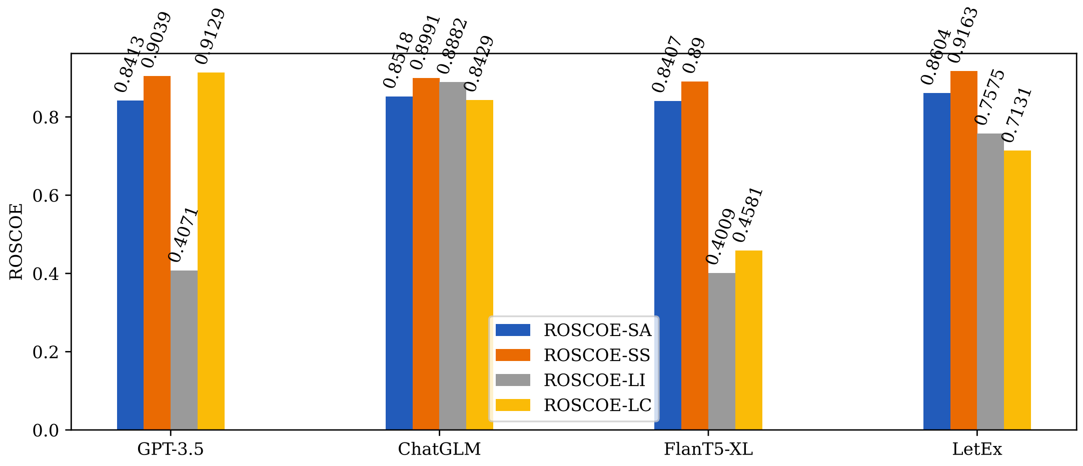

Fig. 7: ROSCOE overall explanation evaluation results on the i2b2 2010 dataset across from 175B GPT-3.5 to 220M LetEx

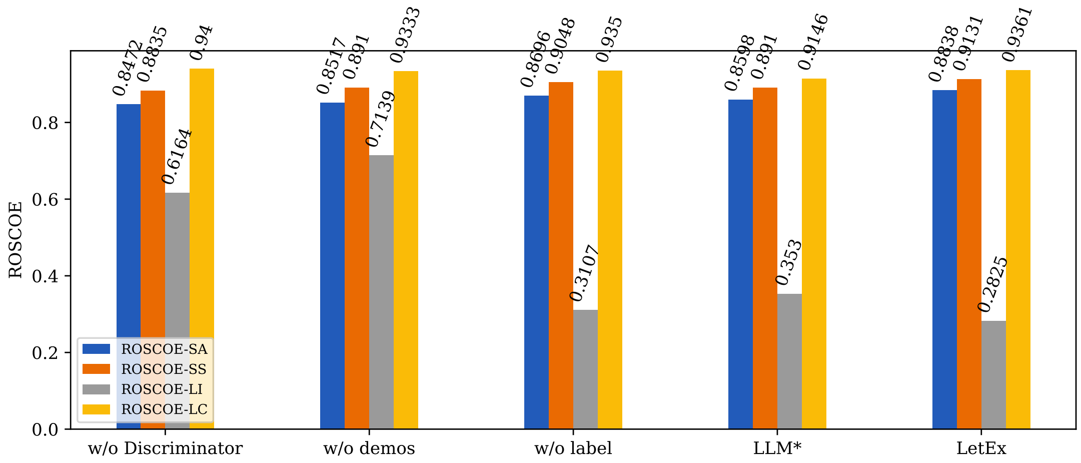

Fig. 8: ROSCOE overall explanation evaluation results on the AIMed dataset under different settings

Table 10. Evaluate the explanation ability of the i2b2 2010 dataset using all ROSCOE metrics. The highest correlation is bolded and the second best is underscored in each row

	175B GPT-3.5-turbo	6B ChatGLM	3B FlanT5-XL	220M LetEx
ROSCOE-SA				
Faithfulness-Step	0.8202	0.8312	<u>0.8407</u>	**0.8456**
Faithfulness-Token	0.8432	<u>0.8518</u>	0.8407	**0.8604**
Info-Step	<u>0.8413</u>	0.8218	0.8208	**0.8453**
Repetition-Token	0.0853	**0.7365**	n/a	<u>0.5151</u>
ROSCOE-SS				
Info-Chain	<u>0.9039</u>	0.8991	0.89	**0.9163**
Repetition-Step	0.0518	**0.7155**	n/a	<u>0.4913</u>
ROSCOE-LI				
Source Consistency	0.4071	<u>0.4605</u>	0.4009	**0.5891**
Self-Consistency	0.1989	**0.8882**	n/a	<u>0.7575</u>
ROSCOE-LS				
Perplexity-Step	0.0076	**0.0081**	0.0004	<u>0.0078</u>
Perplexity-Chain	**0.0544**	0.0208	0.0004	<u>0.0217</u>
Grammar	**0.9129**	<u>0.8429</u>	0.4581	0.7131

O. Golovneva et al. (2023). ROSCOE: A suite of metrics for scoring step-by-step reasoning. In *The Eleventh International Conference on Learning Representations, ICLR 2023, Kigali, Rwanda*.

J. Li et al. (2015). Annotating chemicals, diseases, and their interactions in biomedical literature. In *Proceedings of the fifth BioCreative challenge evaluation workshop*, pages 173–182. The Fifth BioCreative Organizing Committee.

A. Romanov and C. Shivade (2018). Lessons from natural language inference in the clinical domain. In *Proceedings of the 2018 Conference on Empirical Methods in Natural Language Processing, Brussels, Belgium*, pages 1586–1596.

S. Sanyal et al. (2022). Robustlr: A diagnostic benchmark for evaluating logical robustness of deductive reasoners. In *Proceedings of the 2022 Conference on Empirical Methods in Natural Language Processing, EMNLP 2022, Abu Dhabi, United Arab Emirates*, pages 9614–9631.

Ö. Uzuner et al. (2011). 2010 i2b2/va challenge on concepts, assertions, and relations in clinical text. *J. Am. Medical Informatics Assoc.*, **18**(5), 552–556.

T. Wolf et al. (2020). Transformers: State-of-the-art natural language processing. In *Proceedings of the 2020 Conference on Empirical Methods in Natural Language Processing: System Demonstrations, EMNLP*, pages 38–45.

Table 11. Case study of explanations and predicted labels generated by LetEx and other LLMs on the HPRD50 dataset. – signifies no generating explanation

Case 1:<br>**Text**: Cutting edge: @PROTEIN1$ functions as a T cell counterreceptor for the macrophage adhesion receptor @PROTEIN2$ (Siglec-1)<br>**Relation:** PPI	
175B GPT-3.5	**Explation:** Step 1: Identify the two proteins - @PROTEIN1$ and @PROTEIN2$. @PROTEIN1$ = functions as a T cell counterreceptor @PROTEIN2$ = macrophage adhesion receptor (Siglec-1) Step 2: Analyze the relationship between the two proteins. The sentence states that @PROTEIN1$ functions as a counterreceptor for @PROTEIN2$, which means that they are interacting with each other. Step 3: Based on the given relation types, the closest match for this relationship would be “PPI” (protein-protein interaction). Therefore, the final answer is [“PPI”].<br>**Relation:** PPI
6B ChatGLM	**Explation:** Based on the given sentence, the relation between @PROTEIN1$ and @PROTEIN2$ is “PPI”. Note: “Let’s think step by step” is a phrase and not a relation.<br>**Relation:** PPI
3B FlanT5-XL	**Explation:**: False]<br>**Relation:** False
220M LetEx	**Explation:** First, the sentence mentions @PROTEIN1$ working as a T cell counterreceptor for the macrophage adhesion receptor Siglec-1. This implies that @PROTEIN1$ is involved in a protein-protein interaction with another protein. Next, the sentence specifies that this interaction is between @PROTEIN1$ and the PROTEIN2$ (Siglec-1 receptor). This implies that there is a direct protein-protein interaction between @PROTEIN1$ and the Siglec-1 receptor, leading to the “PPI” relation label.<br>**Relation:** PPI
Case 2:<br>**Text**: beta 2 integrin, detected by anti-CD18 mAb, was coprecipitated with @PROTEIN1$ from human neutrophil lysates by a mAb to @PROTEIN2$<br>**Relation:** False	
175B GPT-3.5	**Explation:** Step 1: Identify the two proteins involved in the sentence. - Protein 1: not mentioned in the sentence. - Protein 2: mentioned as the target of a monoclonal antibody (mAb). Step 2: Determine the type of interaction between the two proteins. - The sentence describes that Protein 2 was targeted by a monoclonal antibody and co-precipitated with Protein 1 from human neutrophil lysates. - This suggests a physical interaction between the two proteins, indicating a PPI (protein-protein interaction) relation. Step 3: Final answer - [“PPI”]<br>**Relation:** PIP
6B ChatGLM	**Explation:** False<br>**Relation:** False
3B FlanT5-XL	**Explation:** –<br>**Relation:** False
220M LetEx	**Explation:** The sentence mentions that beta 2 integrin was coprecipitated with @PROTEIN1$ from human neutrophil lysates. It does not provide any evidence of an interaction or relationship between @PROTEIN1$ and @PROTEIN2$. Therefore, the relation label between @PROTEIN1$ and @PROTEIN2$ is “False”<br>**Relation:** False
